# Supplementary material for: Phytochemical composition and bioactivity of Debregeasia saeneb leaves: Insights into anti-diabetic and antioxidant properties
Source: PLoS One. 2025 Jul 2;20(7):e0326991. doi: 10.1371/journal.pone.0326991 (PMC12221095; doi:10.1371/journal.pone.0326991)
Supplement: Attachment — Submitted filename: Editorial and Reviewers comments.docx [file pone.0326991.s003.docx]

**Response to Editor and Reviewers**

**Manuscript ID:** PONE-D-24-51482R1

**Title:** Phytochemical Composition and Bioactivity of *Debregeasia saeneb* Leaves: Insights into Anti-Diabetic and Antioxidant Properties

**Journal:** PLOS ONE

**Dear Editor Dr. Hamida Hamdi Mohammed Ismail**

Thank you for your email and the opportunity to revise our manuscript. We sincerely appreciate the time and effort invested by the academic editor and reviewers in evaluating our work. We are grateful for their constructive feedback, which has helped us improve the quality and clarity of the manuscript.

We have carefully addressed all the comments and suggestions raised during the review process. Below, we provide a point-by-point response to each concern, detailing the revisions made in the manuscript. The changes have been incorporated into:

1. A marked-up copy with tracked changes (uploaded as Revised Manuscript with Track Changes).
2. A clean version of the revised manuscript (uploaded as Manuscript).

**Editorial comments:**

If you would like to make changes to your financial disclosure, please include your updated statement in your cover letter. Guidelines for resubmitting your figure files are available below the reviewer comments at the end of this letter.

Reply: Thank you for your feedback. We have not changed financial disclosure. Additionally, we have make changes according to your instructions (While revising your submission, please upload your figure files to the Preflight Analysis and Conversion Engine (PACE) digital diagnostic tool, [https://pacev2.apexcovantage.com/](https://track.editorialmanager.com/CL0/https:%2F%2Fpacev2.apexcovantage.com%2F/1/010f01962a558548-a575470d-c099-49b7-ba56-90f1aed22809-000000/CXcjXE0z-kva8l4UnFIilX3I85SeZyS94s08SDlsiaQ=206).) and uploaded the figures to the system

**Journal Requirement**

**Comment:**

Please review your reference list to ensure that it is complete and correct. If you have cited papers that have been retracted, please include the rationale for doing so in the manuscript text or remove these references and replace them with relevant current references. Any changes to the reference list should be mentioned in the rebuttal letter that accompanies your revised manuscript. If you need to cite a retracted article, indicate the article’s retracted status in the References list and also include a citation and full reference for the retraction notice.

Reply: Thank you for your comment regarding the reference list. We have carefully reviewed all the cited references and confirm that none of the papers included in our manuscript have been retracted. Therefore, no changes were necessary in the reference list. We appreciate your diligence in ensuring the integrity of the cited literature.

Additional Editor Comments:

-GC figure very bad, the authors should be improved.

Reply: Thank you for your feedback regarding the GC figure. We have improved the quality of the GC figure by enhancing its resolution and clarity to ensure better readability and presentation. We hope the revised figure now meets the journal’s standards.

**Reviewers' comments:**

Reviewer's Responses to Questions

**Comments to the Author**

1. If the authors have adequately addressed your comments raised in a previous round of review and you feel that this manuscript is now acceptable for publication, you may indicate that here to bypass the “Comments to the Author” section, enter your conflict-of-interest statement in the “Confidential to Editor” section, and submit your "Accept" recommendation.

Reviewer #2: All comments have been addressed

Reviewer #5: (No Response)

2. Is the manuscript technically sound, and does the data support the conclusions?

The manuscript must describe a technically sound piece of scientific research with data that supports the conclusions. Experiments must be conducted rigorously, with appropriate controls, replication, and sample sizes. The conclusions must be drawn appropriately based on the data presented.

Reviewer #2: Yes

Reviewer #5: Yes

3. Has the statistical analysis been performed appropriately and rigorously?

Reviewer #2: Yes

Reviewer #5: Yes

4. Have the authors made all data underlying the findings in their manuscript fully available?

The [PLOS Data policy](http://track.editorialmanager.com/CL0/http:%2F%2Fwww.plosone.org%2Fstatic%2Fpolicies.action%23sharing/1/010f01962a558548-a575470d-c099-49b7-ba56-90f1aed22809-000000/c8rQoK1XrlaY3uxwQTLtMo_6l7jg7cIXO8tBEoI0Tn8=206) requires authors to make all data underlying the findings described in their manuscript fully available without restriction, with rare exception (please refer to the Data Availability Statement in the manuscript PDF file). The data should be provided as part of the manuscript or its supporting information or deposited to a public repository. For example, in addition to summary statistics, the data points behind means, medians and variance measures should be available. If there are restrictions on publicly sharing data—e.g. participant privacy or use of data from a third party—those must be specified.

Reviewer #2: Yes

Reviewer #5: Yes

5. Is the manuscript presented in an intelligible fashion and written in standard English?

PLOS ONE does not copyedit accepted manuscripts, so the language in submitted articles must be clear, correct, and unambiguous. Any typographical or grammatical errors should be corrected at revision, so please note any specific errors here.

Reviewer #2: Yes

Reviewer #5: Yes

6. Review Comments to the Author

Please use the space provided to explain your answers to the questions above. You may also include additional comments for the author, including concerns about dual publication, research ethics, or publication ethics. (Please upload your review as an attachment if it exceeds 20,000 characters)

Reviewer #2: The authors have revised the manuscript as per the suggestions and can be accepted in its present form.

**Response:** We are sincerely grateful to Reviewer #2 for their valuable time and insightful feedback on our manuscript. We are delighted that the reviewer found our revisions satisfactory and appreciate their positive assessment of our work. Their constructive suggestions during the initial review round significantly contributed to improving the quality of our manuscript.

Reviewer #5: Dear Editor; The attached article was checked. The manuscript contains interesting information about Phytochemical Composition and Bioactivity of Debregeasia saenab Leaves: Insights into Anti-Diabetic and Antioxidant Properties. **I think that this article is well suited to your journal.**

It is generally good work. The scientific and presentation level of the manuscript is high.
The title is understandable and in line with the text. The text is written in a descriptive and understandable language. The material and method are well described, and adequately detailed Discussion and conclusion are interrelated.

**Comment**: In introduction and ms: Write the name of the authority to the end of the plant’s name. - <http://www.theplantlist.org/> Debregeasia saeneb (Forssk.) Hepper & J.R.I.Wood is an accepted name Please, read the paper and correct them all.

Reply: We are very thankful to the reviewer for their valuable comment. We have added authority to the end of the plant’s name in the revised manuscript

Comment: What is the difference between study and existing studies? Some additions can be made to the key words.

Reply: The study gap has been mentioned at the end of introduction (Line: 130-135). We have also added keywords.

Comment: References were cross-checked.
Reply: Thank you for confirming. We acknowledge that the references have been cross-checked.

Comment: -The paper should be edited according to the writing rules of the journal
Original manuscript. There are, however, a few minor changes required.

Reply: We have changed the whole manuscript according to the journal style.

7. PLOS authors have the option to publish the peer review history of their article ([what does this mean?](https://track.editorialmanager.com/CL0/https:%2F%2Fjournals.plos.org%2Fplosone%2Fs%2Feditorial-and-peer-review-process%23loc-peer-review-history/1/010f01962a558548-a575470d-c099-49b7-ba56-90f1aed22809-000000/hfF9f9zYe-HCdaUbZogGnqMUWBwBlhmJSssruQQYZXM=206)). If published, this will include your full peer review and any attached files.
If you choose “no”, your identity will remain anonymous, but your review may still be made public.
**Do you want your identity to be public for this peer review?** For information about this choice, including consent withdrawal, please see our [Privacy Policy](https://track.editorialmanager.com/CL0/https:%2F%2Fwww.plos.org%2Fprivacy-policy/1/010f01962a558548-a575470d-c099-49b7-ba56-90f1aed22809-000000/DmYb--onlM9orYmWVrRB6LiKcMX9G9hjoOzVRZg1Sr8=206).

Reviewer #2: No

Reviewer #5: No

We believe these revisions have strengthened the manuscript and hope it now meets PLOS ONE’s publication criteria. Thank you again for your consideration. We look forward to your feedback.
