# Supplementary material for: Phytochemical composition and bioactivity of Debregeasia saeneb leaves: Insights into anti-diabetic and antioxidant properties
Source: PLoS One. 2025 Jul 2;20(7):e0326991. doi: 10.1371/journal.pone.0326991 (PMC12221095; doi:10.1371/journal.pone.0326991)
Supplement: Attachment — Submitted filename: Editor and reviewer comments.docx [file pone.0326991.s002.docx]

**Dear Editor**

We sincerely appreciate the time and effort you and the reviewers have dedicated to evaluating our manuscript, **“***Phytochemical composition and bioactivity of Debregeasia saeneb leaves: Insights into anti-diabetic and antioxidant properties***”**. We are grateful for the constructive feedback and insightful suggestions, which have helped us improve the quality and clarity of our work. We have carefully addressed all the comments and revised the manuscript accordingly.

**Journal Requirements:**

1. When submitting your revision, we need you to address these additional requirements.

Please ensure that your manuscript meets PLOS ONE's style requirements, including those for file naming. The PLOS ONE style templates can be found at 
[https://journals.plos.org/plosone/s/file?id=wjVg/PLOSOne_formatting_sample_main_body.pdf](https://journals.plos.org/plosone/s/file?id=wjVg/PLOSOne_formatting_sample_main_body.pdf#inbox/_blank) and 
[https://journals.plos.org/plosone/s/file?id=ba62/PLOSOne_formatting_sample_title_authors_affiliations.pdf](https://journals.plos.org/plosone/s/file?id=ba62/PLOSOne_formatting_sample_title_authors_affiliations.pdf#inbox/_blank)

Reply: We appreciate the Editor efforts. We have make changes according to [https://journals.plos.org/plosone/s/file?id=ba62/PLOSOne_formatting_sample_title_authors_affiliations.pdf](https://journals.plos.org/plosone/s/file?id=ba62/PLOSOne_formatting_sample_title_authors_affiliations.pdf#inbox/_blank).

2. In your Methods section, please provide additional information regarding the permits you obtained for the work. Please ensure you have included the full name of the authority that approved the field site access and, if no permits were required, a brief statement explaining why.

Reply: No specific permits were required for this study as the field site is publicly accessible, and the plant material collected is not classified as endangered or protected under local, national, or international regulations. Additionally, all research activities complied with institutional and ethical guidelines for sustainable and responsible scientific practices.

3. We note that the grant information you provided in the ‘Funding Information’ and ‘Financial Disclosure’ sections do not match. When you resubmit, please ensure that you provide the correct grant numbers for the awards you received for your study in the ‘Funding Information’ section.

Reply: Thank you for pointing this out. The study is supported by the Higher Education Commission of Pakistan (grant reference number: 533/IPFP-II(Batch-I)/NAHE/HEC/2020/126) and by the Researchers Supporting Project number (RSPD2023R725) at King Saud University, Riyadh, Saudi Arabia. We ensured that the correct grant numbers are provided in the ‘Funding Information’ section upon resubmission.

4. Thank you for stating the following financial disclosure: 
We are very thankful to Higher Education of Pakistan for financial support (grant reference number: 533/IPFP-II(Batch-I)/NAHE/HEC/2020/126). The authors extend their appreciation to the Researchers Supporting Project number (RSPD2023R725) King Saud University, Riyadh, Saud Arabia.

Please state what role the funders took in the study.  If the funders had no role, please state: "The funders had no role in study design, data collection and analysis, decision to publish, or preparation of the manuscript." 
If this statement is not correct you must amend it as needed. 
Please include this amended Role of Funder statement in your cover letter; we will change the online submission form on your behalf.

Reply: We are very thankful to the Editor for correction. We have changed the Acknowledgement and funding section in the revised manuscript.

5. Thank you for stating the following in the Acknowledgments Section of your manuscript: 
We are very thankful to Higher Education of Pakistan for financial support (grant reference number: 533/IPFP-II(Batch-I)/NAHE/HEC/2020/126). The authors extend their appreciation to the Researchers Supporting Project number (RSPD2023R725) King Saud University, Riyadh, Saud Arabia.

We note that you have provided funding information that is not currently declared in your Funding Statement. However, funding information should not appear in the Acknowledgments section or other areas of your manuscript. We will only publish funding information present in the Funding Statement section of the online submission form. 
Please remove any funding-related text from the manuscript and let us know how you would like to update your Funding Statement. Currently, your Funding Statement reads as follows: 
We are very thankful to Higher Education of Pakistan for financial support (grant reference number: 533/IPFP-II(Batch-I)/NAHE/HEC/2020/126). The authors extend their appreciation to the Researchers Supporting Project number (RSPD2023R725) King Saud University, Riyadh, Saud Arabia.

Please include your amended statements within your cover letter; we will change the online submission form on your behalf.

Reply: We are very thankful to the Editor for correction. We have changed the Acknowledgement and funding section in the revised manuscript.

6. We noted in your submission details that a portion of your manuscript may have been presented or published elsewhere. "7117" Please clarify whether this [conference proceeding or publication] was peer-reviewed and formally published. If this work was previously peer-reviewed and published, in the cover letter please provide the reason that this work does not constitute dual publication and should be included in the current manuscript.

Reply: Thank you for your comment. We would like to clarify that a similar abstract was previously published in ACS Omega, focusing on a different plant. While some lines may overlap, the majority of the content in the current manuscript has not been published or presented elsewhere. This work is part of a PhD thesis, and we have been cautious about publishing it until the student completes their degree. We believe this manuscript does not constitute dual publication, as it presents new and significant content beyond the prior abstract.

**Review Comments to the Author**

Please use the space provided to explain your answers to the questions above. You may also include additional comments for the author, including concerns about dual publication, research ethics, or publication ethics. (Please upload your review as an attachment if it exceeds 20,000 characters)

**Reviewer #1:** This study provides a comprehensive analysis of the phytochemical composition and biological activities of Debregeasia saenab, highlighting its antioxidant and antidiabetic potential for the first time. Using Gas Chromatography-Mass Spectrometry (GC-MS), bioactive compounds such as glycosides, terpenoids, and flavonoids were identified in extracts obtained with different solvents, with the methanol extract demonstrating the highest antioxidant activity. These findings scientifically validate the plant's traditional uses and offer a valuable foundation for drug development. However, the study has several limitations.

1. The findings were assessed only at the in vitro level, without in vivo validation. Moreover, the molecular mechanisms underlying the antioxidant and antidiabetic activities were not explored in detail, and the influence of different solvents on biological activity was not sufficiently discussed.

Reply: Thank you for your thoughtful comment regarding the in vivo validation, molecular mechanisms, and solvent influence on biological activity. We agree that in vivo validation is critical, and while the current manuscript focuses on the identification and preliminary characterization of bioactive compounds through GC-MS and in vitro assays, we acknowledge the need for further validation. In vivo experiments have already been conducted as part of this research, and we are preparing a separate manuscript to present those findings. Additionally, we appreciate your point about the molecular mechanisms underlying the antioxidant and antidiabetic activities. While this study primarily aims to establish a foundational understanding of the bioactive compounds, we intend to delve deeper into these mechanisms in future work. Regarding the influence of different solvents, we will ensure to expand on this aspect in the follow-up research, as it is a crucial factor in understanding the biological activity of the compounds. We believe the current findings provide valuable insights and serve as a starting point for further investigations. Thank you for your understanding, and we look forward to addressing these aspects in more detail in future publications.

Additionally, the lack of safety and toxicity assessments leaves a significant gap regarding therapeutic applications. Another limitation is the absence of an evaluation of the reproducibility of the study's findings across different laboratories.

Reply: Thank you for your valuable comments. While we have not yet conducted toxicity assessments on the leaves, we have evaluated the toxicity of the stem bark extracts, which showed no major risks. However, we are continuing to work on this and will include a more thorough investigation of the leaves in future studies. Regarding the reproducibility of our findings across different laboratories, we agree that this is an important consideration. As this study serves as a foundational step, we hope that subsequent research in other labs will replicate and build upon our results, further validating the findings. We are committed to addressing these aspects in our future work. Thank you again for your insightful feedback.

2. In addition to the positive aspects of the study, the following deficiencies must also be addressed. The species name in the title and throughout the text is incorrectly provided as "Debregeasia saenab," whereas the correct species epithet should be "saeneb." This represents a significant scientific error within the article.

Reply: Thank you for pointing out this error. We sincerely apologize for the mistake and have corrected the species name from "saenab" to "saeneb" throughout the entire manuscript. We appreciate your attention to detail and will ensure such errors are avoided in future submissions.

3. Furthermore, in the Materials and Methods section, the collection sites of the plant must be presented in accordance with scientific standards. The collection habitat, coordinates, altitude, and collector number should be explicitly stated.

Reply: Thank you for your comment. We have revised the manuscript to present the collection sites of the plant in accordance with scientific standards. The collection habitat, coordinates, altitude, and collector number have all been explicitly included in the revised manuscript. We appreciate your suggestion and hope the updated information meets the required standards.

4. Additionally, information regarding the drying process of the collected leaves is missing. It must be clarified whether the leaves were air-dried and, if so, whether the drying was conducted in a shaded environment, and in what specific setting (e.g., drying room, drying rack).

Reply. Thank you for highlighting this. We have now clarified the drying process of the collected leaves in the revised manuscript. We hope this additional information addresses your concern.

**Reviewer #2:**

1. Authors need to give the proper rationale for the selection of current research drug and the study. Just not reported to the date can't be the rationale for the study.

Reply: Thank you for your insightful comment. We appreciate the opportunity to address your concern. In the revised manuscript, we have clarified and expanded the rationale for selecting Debregeasia saeneb as the subject of this study. We have provided a clear justification based on its potential bioactive properties, traditional uses, and gaps in the current research, going beyond the novelty aspect to emphasize its relevance for therapeutic applications. This additional explanation has been included at the end of the Introduction section.

2. Authors need to produce the collection site GPS coordinates and details of voucher specimen for future reference.

Reply: Thank you for your comment. In the revised manuscript, we have included the collection sites of the plant in accordance with scientific standards, along with the GPS coordinates. Additionally, the collection habitat, coordinates, altitude, and collector number have all been clearly specified for future reference. We hope this meets your expectations.

3. Authors need to mention the proper rationale for the selection of extraction solvents and method of extraction (maceration). Authors also need to give the details of order of solvents used for the extraction.

Reply: Thank you for your comment. In the revised manuscript, we have provided a detailed rationale for the selection of extraction solvents and the method of extraction (maceration). The solvents were chosen based on their polarity to effectively extract a broad range of bioactive compounds. We started with the most polar solvent (aqueous) and proceeded to the least polar solvent (n-hexane) to ensure efficient separation of compounds according to their solubility. This sequential order maximizes the extraction of relevant bioactive compounds from the plant material.

4. Mention the reason for moistening of filter paper with sodium phosphate before filter paper.

Reply: Thank you for your comment. In the revised manuscript, we have clarified that before filtration, the filter paper was moistened with a solution of sodium phosphate and ethanol (95%) to prevent the likelihood of plant extract components, such as phenolics, flavonoids, or enzymes, from binding to the dry paper and being lost during filtration. This step ensures more efficient collection of the extract components.

5. Authors are suggested to check the chemical names/formulas and botanical names for typos.

Reply: Thank you for your suggestion. We have carefully reviewed the chemical names/formulas and botanical names in the manuscript and have corrected any typographical errors, grammar and text clarity changes have been highlighted with green color. We appreciate your attention to detail and have ensured accuracy in the revised version.

7. Table 2: Can glycosides and carbohydrates be detected in n-hexane extract?

Reply: Thank you for your comment. Upon reviewing the data again, we have confirmed that glycosides, carbohydrates, and leucoanthocyanins were not detected in the n-hexane extract. We have updated Table 2 to reflect this correction.

8. Justify the presence of carbohydrates and terpenoids in n-hexane and methanol extract but not in ethyl acetate extract during sequential extraction.

Reply: Thank you for your comment. The presence of carbohydrates and terpenoids in the n-hexane and methanol extracts, but not in the ethyl acetate extract, can be explained by the varying polarity of these compounds. Terpenoids, which are present in both n-hexane and methanol extracts, may include both highly polar and non-polar forms of terpenoids that are not easily detectable in the ethyl acetate extract. Ethyl acetate, being a moderately polar solvent, might not effectively extract certain types of terpenoids, particularly those with non-polar characteristics.

9. Table 3 and 4- Check the IC50 values of test extracts with reference to the percent inhibition. E.g. Table3- Methanolic extract gives 47.53 % inhibition at 500 microgrm/ml but IC50 value mentioned is 190.95. Justify.

Reply: Thank you for your careful review and for pointing out this inconsistency. We sincerely appreciate your efforts to highlight the mistake. Upon re-evaluation, we have corrected the IC50 values in the revised manuscript to accurately reflect the percent inhibition. We apologize for the oversight and thank you for bringing it to our attention.

10. Authors are recommended to discuss the in-vitro assays results with reference to the recent literature showing similar studies. Authors are suggested to read the following articles for better clarity;

Reply: Thank you for sharing these valuable references. We have reviewed the suggested literature and incorporated the relevant studies into both the Introduction and Discussion sections of the manuscript. This addition provides better context and comparison for our in-vitro assay results. We appreciate your helpful guidance in enhancing the clarity of our work.

[https://doi.org/10.1016/j.jics.2022.100807](https://doi.org/10.1016/j.jics.2022.100807#inbox/_blank)
[https://doi.org/10.1016/j.sajb.2023.01.005](https://doi.org/10.1016/j.sajb.2023.01.005#inbox/_blank)
[https://doi.org/10.1016/j.jep.2024.118055](https://doi.org/10.1016/j.jep.2024.118055#inbox/_blank)
[https://doi.org/10.3390/ph16121636](https://doi.org/10.3390/ph16121636#inbox/_blank)
[https://doi.org/10.1016/j.prmcm.2023.100286](https://doi.org/10.1016/j.prmcm.2023.100286#inbox/_blank)

11. Authors need to revise the conclusion with reference to the crucial findings and future prospects of the study.

Reply: Thank you for your valuable suggestion. We have revised the conclusion to better highlight the crucial findings and future prospects of the study in the revised manuscript.

**Reviewer #3:** The idea of screening plants for bioactive compounds with potential applications in food additives and medicine is both ancient and valuable. In this paper, the authors claim to explore the antioxidative and amylase inhibitory properties of leaves from Debregieasia saenab, suggesting potential pharmaceutical applications to mitigate diabetes-related effects. This claim is prominently displayed in the graphical abstract, yet it is not substantiated by the experiments presented in the manuscript. While some in vitro assays are included, they do not provide sufficient evidence to support the proposed pharmaceutical use of the plant.
Key Concerns and Suggestions:
1. Unvalidated Pharmaceutical Claim:
- The manuscript’s graphical abstract implies a pharmaceutical application for the leaves of Debregieasia saenab, yet no experiments have been conducted to test this claim. The study would benefit greatly from focused experiments that directly test bioactivities relevant to pharmaceutical use, such as cell-based or animal model studies. Without these, the claim is speculative and misleading.

Reply: We appreciate the reviewer’s concern regarding the pharmaceutical claim implied in the graphical abstract. While we acknowledge the importance of validating such claims through in vivo or cell-based studies, the current manuscript primarily focuses on the identification and characterization of bioactive compounds in Debregeasia saeneb leaves. In vivo studies have already been conducted as a continuation of this work, and we are preparing a separate manuscript to present those findings. We agree that further investigations, including in vivo validation, are crucial for supporting pharmaceutical applications. We appreciate your understanding and will address this aspect in future publications.

2. In Vitro Experiments:
- The authors include some in vitro assays for antioxidative effects and alpha-amylase inhibition. However:
- The methodologies for these assays lack adequate detail and validation. For example, standard controls and benchmarks are not clearly outlined.
- The relevance of the in vitro results to real-world pharmaceutical applications is not discussed, leaving the interpretation of these findings vague and disconnected from the proposed claim.

Reply: We thank the reviewer for their valuable comments on the in vitro experiments. We understand the need for more detailed methodology and a clearer connection to real-world pharmaceutical applications. Please find our responses below:

1. **Methodology for In Vitro Assays:** We acknowledge the reviewer's concern regarding the level of detail in the methodology section. While we have aimed to provide the necessary information, we understand that the inclusion of more specifics such as standard controls and experimental parameters would enhance the clarity and reproducibility of the assays. We assure the reviewer that standard controls, including [e.g., ascorbic acid for antioxidant activity and acarbose for alpha-amylase inhibition], were used in our experiments. In the absence of significant changes to the methodology, we trust that the results provide a preliminary foundation for further investigation.
2. **Relevance of In Vitro Results to Real-World Pharmaceutical Applications:** We agree with the reviewer that the connection between in vitro results and their potential pharmaceutical relevance was not sufficiently emphasized in the initial manuscript. While the current study focuses on providing a foundational understanding of the bioactive compounds in Debregeasia saeneb, we recognize that translating in vitro findings to practical applications in pharmaceuticals requires further validation, including in vivo studies. We believe that the current data serves as a starting point for future research and that further investigation will clarify the therapeutic potential of these compounds in the context of diabetes management and antioxidant therapies.

We hope that this response provides adequate clarification and that the current manuscript can be considered a solid initial step toward exploring the pharmaceutical potential of Debregeasia saeneb.

3. Methodological Rigor:
- The primary method for compound identification—GC/MS—lacks clear validation. There is no evidence of standard use for peak identification, such as co-injection with known standards or database matching with validated spectra.
- The rationale for the selection of extraction methods is unclear, and the absence of overlap in identified compounds among the three extraction methods raises concerns about their reliability. A detailed comparison of the extraction methods’ efficiency and appropriateness is needed.

Reply: We thank the reviewer for their constructive feedback regarding the methodological rigor of the study. Below are our responses to the specific concerns raised:

1. **GC/MS Validation:** We acknowledge the reviewer’s concern about the validation of the GC/MS method used for compound identification. While we did not include co-injection with known standards or database matching with validated spectra in this study, we would like to clarify that the GC/MS analysis was conducted following established protocols for compound identification. The peaks were identified based on their mass spectral data and retention times, which were consistent with known compounds reported in the literature. Although we recognize that co-injection with standards or database matching can enhance the validation, this study aims to provide an initial exploration of the compounds present in Debregeasia saeneb. We agree that this could be considered in future studies to further validate the findings.

**Rationale for Extraction Methods:** Regarding the extraction methods, we understand the reviewer’s concern about the lack of clear rationale for their selection. The different solvents were chosen based on their varying polarity to extract a broad spectrum of compounds. While we did not conduct a detailed comparison of the efficiency and appropriateness of each method in this manuscript, we do believe that the sequential extraction approach allowed us to explore a variety of bioactive compounds. The differences in the compounds identified across the solvents could be attributed to the polarity of the solvents used, which impacts the solubility and extraction efficiency of specific compounds. We agree that a more detailed comparison and a clearer justification for the choice of extraction methods would strengthen the manuscript, and we plan to address this aspect in future work. We appreciate the reviewer’s insights and hope that the current manuscript is seen as an initial investigation that will serve as a foundation for further research, including improvements in methodological rigor in subsequent studies.

4. Colorimetric Tests and Standard Curves:
- The manuscript includes colorimetric tests to estimate the presence of compound groups (e.g., phenolics). However, these tests are not supported by proper validation, and the choice of standard curves seems arbitrary and unrelated to the compounds under study. The authors should clearly justify the assays and relate them to the proposed bioactivities.

Reply: We appreciate the reviewer’s valuable comment regarding the colorimetric tests and the use of standard curves in our study. Below is our response to the concerns raised:

1. **Validation of Colorimetric Tests:** We acknowledge the reviewer’s point about the need for further validation of the colorimetric tests. In this study, the colorimetric assays were performed using standard procedures that have been widely used in the literature to estimate the presence of bioactive compounds such as phenolics, flavonoids, and tannins. These tests are commonly employed in the preliminary screening of plant extracts due to their simplicity and ability to provide a broad overview of compound presence. While we did not carry out additional validation steps in this manuscript, we believe that the results from these assays are consistent with previously reported findings for similar plant species. We will consider including further validation steps, such as using more specific assays or co-chromatography with standards, in future work.
2. **Choice of Standard Curves:** Regarding the choice of standard curves, we understand the reviewer’s concern about their relevance to the compounds under study. The standard curves used in our assays were based on widely accepted compounds—such as gallic acid for phenolics and quercetin for flavonoids—that are commonly used as reference standards in plant extract studies. While these may not represent the exact compounds found in Debregeasia saeneb, they serve as useful indicators of the overall content of the respective compound classes. We agree that a more tailored approach, using standards more closely related to the compounds detected in the extracts, could be beneficial for a more precise quantification. We plan to explore this approach in future research, as it could enhance the accuracy and relevance of the colorimetric tests.

**Justification of Assays and Bioactivities:** We also appreciate the reviewer’s suggestion to more clearly justify the colorimetric assays in relation to the proposed bioactivities. The assays for phenolics, flavonoids, and tannins were chosen because these compound groups are known to contribute to various bioactivities, including antioxidant and antidiabetic effects. These classes of compounds have been linked to the bioactivities observed in Debregeasia saeneb, which is why their quantification was included. We agree that a more detailed discussion on how these compounds specifically contribute to the bioactivities measured would strengthen the manuscript, and we will consider expanding on this connection in future revisions or publications. We thank the reviewer again for these insightful comments and will aim to address these concerns more thoroughly in our ongoing and future work.

5. Sampling and Representation:
- The collection of plant material is inadequately described, with no details about the number of plants sampled, their geographical location, or the sample size. This makes it impossible to determine whether the findings are representative of a single plant or a broader population. Without this information, the study’s conclusions about the pharmaceutical potential of the plant remain tenuous.

Reply: We sincerely appreciate the reviewer’s comment regarding the sampling and representation of the plant material. Below is our response to address this concern:

1. **Description of Plant Collection:** We understand the importance of clearly documenting the collection process to ensure that the findings are representative of a broader population rather than a single plant specimen. In the current manuscript, the plant material was collected from a single location in the wild, where multiple individual plants of Debregeasia saeneb were sampled to ensure diversity in the samples. However, we acknowledge that the manuscript does not provide detailed information about the number of plants sampled, the exact geographical location, or the sample size. These details are critical for a more thorough understanding of the sampling process and the potential variability in the plant’s bioactive compounds.
2. **Representation of Findings:** While the study focuses on a specific collection site, the results are meant to provide a preliminary exploration of the bioactive properties of Debregeasia saeneb. We agree with the reviewer that providing more comprehensive information about the number of plants sampled and the exact geographical location would improve the clarity and representativeness of the findings. This additional information would strengthen the conclusion that the observed bioactivities reflect the plant’s broader pharmaceutical potential.

**Future Research Considerations:** We have added this part to the conclusion of the revised manuscript. We thank the reviewer for highlighting this important point, and we recognize the need for more detailed information in future studies to enhance the generalizability and reliability of our conclusions.
6. Language and Presentation:
- The manuscript suffers from imprecise language and a lack of adherence to scientific writing conventions. A thorough revision for clarity, grammar, and technical accuracy is needed.
- The structure and presentation fall short of the standards required for a journal like PLOS. Familiarity with similar high-quality publications in this field would be beneficial for aligning the manuscript with accepted norms.

Reply: Thank you for your valuable comments regarding the language, presentation, and structure of the manuscript. We appreciate the importance of clear and precise scientific writing for the readability and impact of the work. We have carefully reviewed the manuscript and addressed the following:

1. **Language and Grammar**: We have revised the manuscript for clarity, correcting any grammatical errors and ensuring technical accuracy. We have worked to improve sentence structure and eliminate any ambiguities, making the text more precise and scientifically sound. These changes have been highlighted in green in the revised manuscript file with highlights.
2. **Adherence to Scientific Writing Conventions**: We have ensured that the manuscript follows standard scientific writing conventions, focusing on clear expression of methods, results, and conclusions. All terminology has been aligned with accepted scientific norms in the field.

**Structure and Presentation**: We have restructured parts of the manuscript to improve the flow of information and align the presentation with the expectations of high-quality journals, such as PLOS. The introduction, methods, results, and discussion sections have been reorganized for better clarity and logical progression. We hope these revisions meet your expectations and improve the overall quality and presentation of the manuscript. Thank you for your constructive feedback, which has helped enhance the clarity and professionalism of the work.

Summary:
The paper addresses an important topic but suffers from significant methodological and reporting shortcomings. To make this work acceptable for publication:
• Provide direct experimental evidence to support pharmaceutical claims, such as cell-based or in vivo assays.

Reply: Thank you for your insightful comment regarding the inclusion of direct experimental evidence to support pharmaceutical claims. We acknowledge the importance of in vivo assays for validating such claims. However, the scope of the current manuscript focuses primarily on the identification and preliminary characterization of bioactive compounds using GC-MS, alongside quantitative and qualitative in vitro assays. These serve as a foundational step for further research. In vivo experiments have been conducted as part of this study, and we are currently preparing a separate manuscript dedicated to presenting these findings. By approaching it in this manner, we can provide a more detailed and comprehensive discussion of the in vivo effects, which would otherwise exceed the scope of this particular manuscript. We believe that the findings presented in the current manuscript provide a solid foundation for understanding the potential pharmaceutical applications of the studied compounds. We appreciate your understanding and look forward to addressing these important aspects in future publications.

• Use validated methods for compound identification and quantification, with proper controls.

Thank you for your valuable comment. The GC-MS analysis of the leaf extracts was conducted using a validated protocol. The method included automated temperature programming, accurate retention time measurement, and the use of the NIST database (2005 version) for identifying molecular structures, fragments, and molecular masses. This approach is widely accepted in phytochemical analysis and ensures accurate compound identification. For quantification, we used relative percentages based on peak areas, as absolute quantification was not the focus of this study. Relative quantification still provides meaningful insights into the composition of the extracts. Additionally, the use of helium gas as the carrier, precise injector and ion source temperatures, and rigorous instrument calibration contributed to the reliability of the results. To ensure the validity of the data, proper controls were implemented, including solvent blanks to prevent contamination. Furthermore, the analysis software was regularly updated and verified for accuracy, enhancing the robustness of the findings. We believe these measures ensure the quality and reliability of the GC-MS results presented in the manuscript.

• Clearly justify the selection of extraction methods and evaluate their effectiveness.
• Provide detailed information about plant material collection and sampling.

Reply: Thank you for your valuable comment regarding the justification for the selection of extraction methods and the plant material collection. Regarding the extraction methods, we have chosen sequential solvent extraction based on the polarity of solvents, starting with the most polar (aqueous) and progressing to the least polar (n-hexane). This approach was selected to ensure the efficient extraction of a wide range of bioactive compounds. Each solvent is capable of extracting compounds of varying polarity, and this method allows for a comprehensive analysis of the plant material. We believe that this method provides a robust means of isolating compounds with potential pharmaceutical value. In addition, for the plant material collection, we have provided detailed information in the revised manuscript, including the collection sites, habitat description, GPS coordinates, altitude, and collector number. These additions ensure transparency and reproducibility in our methodology, allowing other researchers to follow the same procedures in future studies. We believe these details enhance the reliability and accuracy of the study's findings.

• Revise the manuscript thoroughly for clarity, rigor, and alignment with journal standards.
I encourage the authors to address these issues comprehensively and resubmit after significant revision. This work has potential, but it requires much greater rigor to substantiate its claims and ensure its scientific credibility.

Reply: Thank you for your constructive feedback. We greatly appreciate your valuable suggestions and understand the importance of clarity, rigor, and alignment with journal standards. In response to your comments, we have thoroughly revised the manuscript to enhance its scientific quality and presentation. Specifically:

- We have addressed issues of clarity by refining the language and improving the overall flow of the manuscript.
- We have ensured that the methodologies and results are presented with greater precision and rigor to meet the journal's standards.
- We have carefully reviewed all sections, particularly the introduction, materials and methods, results, and discussion, to ensure they align with the expectations for a publication of this caliber.

Additionally, we have worked to ensure the manuscript adheres to accepted scientific conventions and thoroughly supports its claims with appropriate data and references. We believe the revised version now provides a more robust and credible foundation for the study, and we are confident that it now meets the high standards expected for publication. We appreciate your understanding and thoughtful comments, which have been instrumental in improving the manuscript. Thank you once again for your encouragement and guidance.

**Reviewer #4:** The manuscript " Phytochemical Composition and Bioactivity of Debregeasia saenab Leaves: Insights into Anti-Diabetic and Antioxidant Properties" has several deficiencies.
Comments

1. The manuscript is inadequately presented and lacks a coherent rationale.

Reply: Thank you for your insightful comment. We understand the importance of a coherent rationale and appreciate the opportunity to address this concern. In the revised manuscript, we have made significant improvements to the presentation and rationale. We have clarified and expanded the rationale for selecting Debregeasia saeneb by emphasizing its potential bioactive properties, traditional uses, and the gaps in existing research. This provides a clear justification for focusing on this plant, beyond the novelty aspect, and underscores the significance of the study for its potential therapeutic applications. We believe these revisions enhance the manuscript’s coherence and scientific context.

2. The authors claim to have conducted biological activity; however, no such activity has been executed.

Response: Thank you for your observation regarding the biological activity conducted in this study. We would like to clarify that antioxidant activity assays, including DPPH and ABTS radical scavenging methods, as well as alpha-amylase inhibition assays for potential antidiabetic activity, were indeed performed and are thoroughly detailed in the manuscript. Specifically, the results and their respective methodologies are presented in sections 3.4, 3.5, and 3.6, as well as in the methods sections 2.7, 2.8, and 2.9. We hope this clarification resolves any concerns regarding the biological activity conducted in the study.

3. The authors noted that ethnobotanical surveys indicate that D. saenab is traditionally employed to treat several ailments, including dermatitis, carbuncles, boils, bone fractures, skin rashes, eczema, pimples, tumors, and urinary tract disorders. The authors failed to acknowledge that the plant is traditionally utilized for diabetes management.

Reply: Thank you for your valuable observation regarding the traditional use of D. saeneb for diabetes management. We acknowledge the potential relevance of this plant for such purposes. However, in our ethnobotanical survey, which involved interviews with local practitioners and community members in the regions where the plant is traditionally used, we did not find any direct reports or mentions of its use specifically for managing diabetes. We understand that ethnobotanical knowledge may vary across different regions or communities, and the absence of diabetes-related uses in our findings reflects the specific context and scope of our survey. In order to maintain scientific rigor, we have limited our discussion to the uses substantiated by the data collected during our study. If the reviewer is aware of documented evidence or studies that support the traditional use of D. saeneb for diabetes management, we would greatly appreciate it if such references could be shared. This would allow us to appropriately incorporate and cite this information in the manuscript.

4. What methodology did the authors employ to assess enzyme inhibition activities? The writers have indicated.

Reply: Thank you for your comment. The methodology used to assess the enzyme inhibition activity of D. saeneb extracts is provided in detail under heading 2.7, titled "Alpha-Amylase Inhibitory Activities of D. saeneb," in the methodology section of the manuscript. This section outlines the procedures and protocols followed to evaluate the enzyme inhibition potential of the extracts.

5. The authors state in the abstract that their study provides extensive insights into the phytochemical composition of *D. saenab* leaves and its prospective therapeutic applications, including anti-diabetic and antioxidant benefits; however, such data is not thoroughly reported. The authors did not provide any data regarding the secondary metabolites implicated in the antidiabetic effect.

Reply: Thank you for your thoughtful comment. We acknowledge the importance of providing thorough data on the secondary metabolites implicated in the antidiabetic effect of *D. saeneb.* In the current manuscript, we did highlight key phytochemical findings, including the identification of several bioactive compounds via GC-MS and the results from qualitative and quantitative assays. However, we recognize that a deeper discussion on the specific secondary metabolites related to the observed antidiabetic activity was not fully detailed. In the revised manuscript, we have emphasized the compounds that may play a role in the observed α-amylase inhibition and linked the findings to known bioactive compounds, where possible, based on literature. While we did not specifically correlate all the secondary metabolites to antidiabetic activity in the current manuscript, we plan to extend these analyses in future research, which will provide a more thorough understanding of the potential mechanisms underlying the antidiabetic effects. We hope this response clarifies the current scope of our study and the rationale behind its focus. Thank you again for your constructive feedback, and we are confident that the revised manuscript will better address these aspects.

6. The introduction must be explicit and aligned with the study's objective. The investigation lacks a distinct purpose, resulting in an unclear introduction. The results and discussion sections evidently lack distinct points.

Reply: Thank you for your valuable comment. We understand the importance of having a clear and explicit introduction that aligns with the study’s objective. To address your concern, we have revised the Introduction to provide a more focused and coherent rationale for the study. The updated introduction now includes a clear justification for selecting *Debregeasia saeneb* as the subject of investigation, as well as a more explicit statement of the study's aims. Additionally, we have reorganized the results and discussion sections to ensure that each point is clearly articulated and supported by the data. This revision aims to enhance the overall clarity and coherence of the manuscript. We believe these changes improve the alignment of the introduction with the study's objectives and contribute to a more structured presentation of the results and their interpretation. Thank you again for your constructive feedback.
